# Biomass, Phyto-Ash, and Biochar from Beech Wood as Functional Additives for Natural Rubber-Based Elastomer Composites

**DOI:** 10.3390/ma18071659

**Published:** 2025-04-04

**Authors:** Justyna Miedzianowska-Masłowska, Marcin Masłowski, Krzysztof Strzelec

**Affiliations:** Institute of Polymer and Dye Technology, Lodz University of Technology, Stefanowskiego 16, 90-537 Lodz, Poland

**Keywords:** biocomposites, natural rubber, beech wood, biomass, phyto-ashes, biochar, functional properties

## Abstract

The growing interest in renewable resource-based materials has driven efforts to develop elastomeric biocomposites using biomass, phyto-ash, and biochar as fillers. These bio-additives, derived from beech wood through various processing methods, were incorporated into natural rubber (NR) at varying weight ratios. The primary objective of this study was to assess how the type and content of each bio-filler influence the structural, processing, and performance properties of the biocomposites. Mechanical properties, including tensile strength and hardness, were evaluated, while crosslink density of the vulcanizates was determined using equilibrium swelling in solvents. Additionally, the composites underwent thermogravimetric analysis (TGA) to determine the decomposition temperature of individual components within the polymer matrix. Bio-fillers influenced rheological and mechanical properties, with phyto-ash reducing viscosity and cross-linking density, and biochar and biomass increasing stiffness and maximum torque. Biochar extended curing time due to the absorption of curing agents, whereas phyto-ash accelerated vulcanization. Mechanical tests showed that all bio-filled composites were stiffer than the reference, with biochar and biomass (30 phr) exhibiting the highest hardness (45.8 °ShA and 49.1 °ShA, respectively) and cross-link density (2.68 × 10^−5^ mol/cm^3^ and 2.77 × 10^−5^ mol/cm^3^, respectively), contributing to improved tensile strength, in particular in the case of biochar, where the TS was 17.6 MPa. The study also examined the effects of thermal-oxidative aging on the samples, providing insights into the changes in the mechanical properties of the biocomposites under simulated aging conditions.

## 1. Introduction 

Biocomposites are materials made of two or more components, with at least one being biologically derived. These materials often combine natural polymers (e.g., proteins) or synthetic ones (e.g., lactic acid-based polymers) with reinforcements from sources like plant fibers (e.g., flax) or minerals (e.g., silica) [1,2,3,4]. In such composites, natural elements act as fillers or reinforcements, while synthetic polymers form the matrix, creating materials with distinctive properties [5,6].

Biocomposites are attractive due to their biodegradability, reducing waste and landfill pressure, and are often sourced sustainably, minimizing reliance on synthetic resources [7,8]. Additionally, their production can yield lower greenhouse gas emissions compared to traditional materials, as biomass or biodegradable polymer-based processes are often less energy-intensive [9,10]. These advantages have led to growing applications in fields like materials engineering, medicine, packaging, and the automotive sector [11,12,13].

Biobased fillers, sourced from natural or ecological origins, offer sustainable alternatives to traditional synthetic fillers like carbon black or silica, enhancing product eco-friendliness [14,15]. Common examples include plant fibers (cotton, flax, hemp, and bamboo) [16,17,18], wood products (wood chips and flour) [19,20], or starch [21]. Natural fillers in biocomposites, while challenging, offer substantial environmental benefits, promoting biodegradability and a reduced ecological footprint [22]. Research continues to optimize their application in biocomposites [23].

The incorporation of beech wood as a filler in elastomers can significantly enhances the mechanical performance and durability of polymer composites. Due to its intrinsic fibrous structure and high cellulose content, beech wood improves stiffness, tensile strength, and wear resistance by reinforcing the polymer matrix and promoting effective stress transfer [24,25]. Its natural porosity facilitates better interfacial adhesion, contributing to improved load-bearing capacity and impact resistance. Moreover, as a biodegradable and renewable material, beech wood supports the development of sustainable biocomposites, offering an eco-friendly alternative to synthetic fillers while potentially reducing production costs. Its adaptability in various forms, such as fibers, chips, or powder, further expands its applicability in elastomeric systems, enabling tailored modifications to thermal insulation, elasticity, and overall composite performance [26].

The use of beech wood as a filler in natural rubber presents a promising research direction, offering a balance between mechanical performance and environmental sustainability. However, the method of incorporating this bio-additive into elastomeric materials plays a crucial role. Integrating plant-based waste materials, such as ash, biomass, and biochar, into polymer technology appears to be a viable strategy for enhancing both the sustainability and performance of composite materials. Ash, often regarded as a byproduct of combustion processes, is rich in minerals and can serve as a filler to improve the mechanical properties and thermal stability of polymer matrices. These ashes provide benefits such as increased stiffness and reduced weight, while contributing to the overall sustainability of the composite by reusing waste materials that would otherwise be disposed of in landfills [27,28].

Biomass, encompassing a wide range of organic materials, serves as a renewable resource that can be transformed into biopolymers or utilized as fillers in conventional polymers. The incorporation of biomass into polymer matrices not only enhances their mechanical properties but also offers potential biodegradability, aligning with the increasing demand for eco-friendly materials [29]. Furthermore, biomass-derived fillers can contribute to improved impact resistance and enhance the thermal and acoustic insulation properties of the resulting composites [30].

Biochar, a carbon-rich material derived from biomass pyrolysis, has become a valuable additive in polymer formulations. Its distinctive porous structure and large surface area contribute to enhanced mechanical and thermal properties of polymer composites while also offering additional advantages, such as increased resistance to moisture and microbial degradation [31]. The use of biochar can also improve the fire resistance of polymers, thereby expanding the range of applications in which these composites can be effectively utilized [32,33].

The incorporation of ash, biomass, and biochar enhances resource efficiency and minimizes the environmental footprint of polymer production. These sustainable fillers enable the development of high-performance elastomeric composites that align with both functional and ecological standards. This research aims to contribute to a more sustainable future in materials science by optimizing bio-based composite formulations.

## 2. Materials and Methods

### 2.1. Preparation of Bio-Fillers

Bio-fillers for rubber composites were sourced from waste beech wood shavings. (bulk density—approx. 200 kg/m^3^, shape—irregular, humidity—approx. 10%, color—naturally light brown). Biomass (BM) was obtained by mechanically processing dried shavings in a ball mill for 30 min, yielding a particle size suitable for composite applications.

Biochar (BC) and phyto-ash (PA) were prepared through thermal treatment of the beech wood shavings. To produce biochar, the milled biomass (70 g) was placed in a custom crucible under a nitrogen gas flow of 200 mL/min to ensure an inert atmosphere by preventing oxygen ingress. The crucible was heated at 600 °C for 2 h, after which the furnace was powered down, and the material was allowed to cool under nitrogen flow for an additional 2 h. In the case of phyto-ash production, the thermal treatment process was carried out without the use of inert gas. The ground biomass was subjected to high temperature treatment in an oxygen atmosphere at 600 °C and for a period of 2 h. The conditions for conducting the pyrolysis process to obtain biochar were determined based on our previous research [33].

### 2.2. Composition of the Mixtures

The compositions of the mixtures for further tests are presented in Table 1. The rubber mixtures were prepared, one without filler, and the rest contained produced bioad-ditives in the form of wood flour/biomass, biochar, and phyto-ash in various parts by weight.

### 2.3. Preparation of Rubber Mixtures

The mixtures were prepared in two stages. In the first stage, the elastomer was combined with the filler in a laboratory micromixer (Brabender, Duisburg, Germany), rotor speed 45 rpm, chamber temperature 60 °C, mixing time 15 min. Then, a crosslinking unit (Sulfur—5 phr, Zinc oxide (ZnO)—2 phr, mercaptobenzothiazole (MBT)—2 phr, Stearate—1 phr) was added to each mixture on a laboratory two-roll mill (Bridge, UK). Process parameters: roll dimensions: D = 140 mm, L = 300 mm; the rotational speed of the front roll: Vp = 16 min^−1^; the friction and the width of the gap between rollers: 1–1.2, 1.5–3 mm; the average temperature of the rolls: about 30 °C.

### 2.4. Rheometric Properties of the Compounds

The rheometric properties of the elastomer mixtures were determined using an Alpha Technologies rotorless rheometer at a temperature of 160 °C. The test consisted of recording the torque value as a function of vulcanization time. Then, the value of the torque increase during vulcanization was determined (Equation (1)). The time of vulcanization and pre-vulcanization was also determined based on the obtained rheometric curves.

ΔG = G_max_ − G_min_ (1)

ΔG—increase in rotor torque [dNm];

G_max_—maximum value of rotor torque [dNm];

G_min_—minimum value of rotor torque [dNm].

### 2.5. Determination of Vulcanization Kinetics

The kinetics of vulcanization in elastomer mixtures with bio-additives were analyzed via differential scanning calorimetry (DSC) using a DSC calorimeter (Mettler Toledo, Greifensee, Switzerland). Rubber blend samples (8–9 mg) were hermetically sealed in calorimetric crucibles, cooled to −100 °C at a rate of 10 °C/min, and subsequently heated from −100 °C to 250 °C at a rate of 10 °C/min. Nitrogen at a flow rate of 20 mL/min was used as the protective gas.

### 2.6. Scaning Electron Microscopy Analysis (SEM)

SEM images of bioadditives and NR composites were taken using an LEO1450 SEM microscope (Carl Zeiss AG, Oberkochen, Germany). Prior to the measurement, vulcanizates were broken down using liquid nitrogen; their fractures were coated with carbon and next examined.

### 2.7. Crosslinking Density of the Vulcanizates

The crosslinking density of vulcanizate samples was determined following the PN-ISO 1817:2021 standard [34], using the equilibrium swelling method. This measurement is based on assessing the mass change in samples exposed to a solvent, in this case, toluene. From each vulcanizate, four specimens were cut to achieve a mass range of 20–50 mg. The samples were placed in a weighing vessel, submerged in the solvent, and allowed to swell for 48 h. After this period, the samples were weighed, then dried in an oven at 50 °C for 48 h. The dried samples were then reweighed. This procedure allowed for the determination of the following parameters [35]:

- Equilibrium Swelling, *Q_w_* (Equation (2)):

(2) $$Q_{w}=\frac{m_{sp}-m_{s}}{m_{s}}$$

where

*Q_w_* is the equilibrium swelling;

*m_sp_* is the mass of the swollen sample;

*m_s_* is the mass of the dried sample.

- Concentration of Effective Chains [36], $\gamma_{e}$ (Equation (3)):

(3) $$\gamma_{e}=\frac{\ln\left(1-V_{r}\right)+V_{r}+\mu V_{r}^{2}}{V_{0}(V_{r}^{\frac{1}{3}}-\frac{V_{r}}{2})}$$

where *V_r_* is the volume fraction of rubber in the swollen gel (Equation (4)):

(4) $$V_{r}=\frac{1}{1+Q_{w}\frac{\rho_{k}}{\rho_{r}}}$$

where

*γ*_e_ is the effective crosslink density [mol/cm^3^];

*V_r_* is the volume fraction of rubber in the swollen gel;

*ρ_k_* is the density of the rubber [g/cm^3^];

*ρ_r_* is the density of the solvent [g/cm^3^];

*V*_0_ is the molar volume of the solvent [mol/cm^3^];

*μ* is the Huggins interaction parameter for the elastomer-solvent interaction at 25 °C (Equation (5)):

μNR + toluene = 0.487 + 0.228V_r_ (5)

where

μNR + toluene—the Huggins interaction parameter for the natural rubber (NR)–toluene interaction at 25 °C [37].

### 2.8. Mechanical Properties of Vulcanizates Under Static Conditions

The mechanical properties of the vulcanizates were evaluated in accordance with the PN-ISO 37:2007 standard [38] using a tensile testing machine (Zwick, Ulm, Germany), model 1435. Five paddle-shaped type w-3 specimens were cut from each vulcanizate, with a gauge section width of 4 mm. The thickness of the gauge section was measured at three points, with the average value used for testing. The average values were presented alongside their standard deviation, and a one-way analysis of variance (ANOVA) was performed. The statistical analysis revealed a significant difference in the mechanical properties of composites with different fillers (*p* < 0.05). Each specimen was then mounted on the testing machine.

This study enabled the determination of the following parameters:

SE_100_, SE_200_, SE_300_ [MPa]: stress at 100%, 200%, and 300% elongation;

TS [MPa]: tensile strength, defined as the stress at specimen break;

EB [%]: relative elongation at break.

### 2.9. Determination of Vulcanizates Hardness 

The hardness of selected vulcanizates was determined according to the PN-EN ISO 868:2005 standard [39] using a Shore A durometer (Zwick, Germany). For each disk-shaped sample (~10 mm thickness), six hardness measurements were performed, and the final result was reported as the average of these measurements.

### 2.10. Thermogravimetric Analysis (TGA) of Fillers and Vulcanizates

- Fillers

The analysis was applied to evaluate the thermal stability of natural fillers. Measurements were conducted using a TGA/DSC analyzer (Mettler Toledo, Switzerland), calibrated with standard indium and zinc references. To perform pyrolysis of the natural additives, approximately 10 mg of each sample was placed in an alumina crucible and heated from 25 °C to 600 °C under an argon atmosphere (flow rate of 60 mL/min) at a heating rate of 10 °C/min. In certain cases, the samples were further heated up to 700 °C in an air atmosphere (flow rate of 60 mL/min) to oxidize any remaining decomposition residues. Nitrogen gas with a flow rate of 20 mL/min was used as a protective gas during measurements.

- Vulcanizates

Measurements were performed on approximately 10 mg samples of vulcanizates placed in alumina crucibles. The samples were heated in an argon atmosphere (flow rate of 60 mL/min) from 25 °C to 600 °C at a heating rate of 20 °C/min. Upon reaching 600 °C, the atmosphere was switched to air (60 mL/min), and any pyrolysis residues were combusted by further heating up to 900 °C, with the heating rate maintained.

### 2.11. Thermo-Oxidative Aging

In this study, representative samples of vulcanized rubber were selected, cleaned, and initially characterized by measuring properties such as hardness, tensile strength, elongation at break, crosslinking density, and hydrophobicity (contact angle measurement). The samples were then arranged in a dryer (Binder), ensuring adequate spacing to facilitate uniform air circulation.

The dryer was preheated to a predetermined temperature (70 °C) and the samples were exposed to this environment for 10 days to simulate accelerated thermal-oxidative aging. Throughout the test, temperature and airflow were continuously monitored to ensure stable and reproducible conditions [40].

After the aging period, the samples were re-subjected to functional characterization tests to assess the degree of degradation induced by the thermal-oxidative aging process. Based on the results of mechanical strength, the aging factor (K) was calculated according to Equation (6) [41]:

K = (TS × EB)after aging/(TS × EB)before aging(6)

where

TS—tensile strength [MPa];

EB—elongation at break [%].

### 2.12. Contact Angle (CA) Measurement

A static water drop method was employed to measure the contact angle on vulcanizate surfaces. The contact angle between a water drop (10 μL) and the compressed vulcanizate surface was measured using a goniometer (DataPhysics OCA 15EC, Charlotte, NC, USA) based on images taken immediately after drop placement.

## 3. Results and Discussion

### 3.1. Rheometric Properties of Elastomeric Blends

The initial phase of the study focused on examining the influence of filler type and concentration on the rheometric properties of elastomeric composites (Table 2).

Results indicated that the introduction of phyto-ash slightly reduced the minimum torque value (G_min_), which correlates with a decrease in compound viscosity. This effect is likely due to the high mineral content in the phyto-ash. In contrast, biochar and biomass fillers resulted in a slight increase in G_min_, suggesting that the addition of these rigid phases increases the stiffness of the mixture relative to the reference compound.

Among all biocomposites, the biomass-filled mixtures exhibited the highest maximum torque (G_max_), potentially linked to the inherent consistency of the biomass filler. Notably, the torque increase observed in compounds with biochar and biomass may also imply a higher crosslink density, as G_max_ serves as an indirect indicator of crosslinking density. Conversely, compounds with phyto-ash displayed the lowest G_max_ values, suggesting a comparatively lower crosslink density.

A consistent trend of increased torque was observed across all filler types with higher filler concentrations. Additionally, the vulcanization time, critical for determining the optimal cure time of rubber compounds, was examined. Composites containing phyto-ash and biomass exhibited vulcanization times slightly shorter or similar to the reference sample, whereas biochar-filled compounds demonstrated a marginally prolonged t_90_ value.

Longer vulcanization times observed across all biocomposites, as compared to the reference, may stem from the fillers’ poorer dispersion or their absorptive capacities, which likely retain certain curative components on their surfaces, diminishing crosslinking efficiency. Consequently, biofillers appeared to influence the activity of curative agents during vulcanization, contributing to increased t_90_ values. Phyto-ash-filled composites displayed the shortest vulcanization times, likely due to basic compounds within the ash accelerating the crosslinking process. In contrast, biochar-filled compounds exhibited the longest vulcanization times, potentially due to the mildly acidic nature of biochar, which may delay crosslinking reactions.

### 3.2. DSC Analysis of Elastomeric Compounds

DSC analysis was used to determine several key thermal properties that are important in the design, processing and quality assessment of elastomeric compounds: energy effect, initial and final vulcanization temperature and glass transition temperature (Table 3). Measurements were performed for compounds containing 30 parts by weight of the fillers tested and a reference sample.

DSC analysis results indicated a slight decrease in the glass transition temperature (Tg) for blends with added bio-based additives, likely due to the impact of fillers on the molecular structure and dynamics of the polymer chains [42,43]. The mechanisms underlying this phenomenon are diverse. One contributing factor may be an increase in the mobility of polymer chain segments, where filler particles act as spacers between polymer chains, enhancing the freedom of segmental movement and rendering the material more flexible and elastic at lower temperatures. Additionally, the change in Tg could stem from a reduction in intermolecular interactions; the presence of fillers may limit direct interactions between polymer chains. For non-polar fillers, such as carbon-based fillers, these particles can act as barriers between elastomer chains, reducing van der Waals forces and other intermolecular interactions. Another mechanism potentially affecting Tg of elastomer blends is the plasticizing effect of certain fillers. Some fillers may behave similarly to plasticizers, especially if their chemical properties facilitate interactions with the elastomer matrix. Finally, Tg reduction may also result from structural disruption within the elastomer matrix, as fillers induce local stresses and irregularities that alter the spatial arrangement of polymer chains.

Moreover, it was noticed that the addition of biomass, biochar, and phytogenic ash to rubber compounds increases the enthalpy of vulcanization. This was probably due to the introduction of additional functional groups, such as hydroxyl, carboxyl, and phenolic groups, which interact with polymers in the mixture and increase the number of reactive sites [44,45,46]. These interactions require additional energy, thereby raising the overall enthalpy of the process. Additionally, biochar and phytogenic ash contain metals such as calcium, magnesium, and potassium, which may act as catalysts in the vulcanization process, altering the reaction mechanism and leading to the formation of a greater number of cross-links between polymer chains, thus demanding further energy input. These additives also have high specific surface areas and porous structures, which increase contact surfaces with the polymer and intensify chemical reactions at phase interfaces. The high surface activity and structural irregularity of these additives intensify the reaction processes, which in turn require greater energy input. Accordingly, the more complex spatial cross-linking process raises the enthalpy of vulcanization, as the formation of stable bonds and a high-density structure necessitates significant energy resources. At the same time, it was observed that the effect of using selected bio-additives had a positive effect on reducing the initial temperature of the cross-linking process, which can undoubtedly contribute to improving energy efficiency, product quality and reducing the impact on the environment, as well as to better use of natural and ecological additives.

### 3.3. Scanning Electron Microscopy (SEM) Analysis

To comprehensively assess the morphology and structure of the obtained bio-additives derived from beech wood (including ground biomass, ash, and biochar) as well as the composites incorporating these materials, scanning electron microscopy (SEM) analysis was conducted. The results of this analysis are illustrated in Table 4.

The analysis of SEM images of phyto-ash obtained from the combustion of beech wood revealed the presence of diverse structures, differing in shape, size, and morphology. The observed particles exhibited a range of structural forms, including amorphous, fibrous, crystalline, hybrid, and layered types. Each of these structural variants plays a significant role in the functionality of phyto-ash within composites, influencing interactions with the polymer matrix as well as the material’s mechanical and thermal properties. Amorphous structures displayed a lack of order, which may affect the solubility and flexibility of the composites, while fibrous and crystalline structures could contribute to material reinforcement due to their specific mechanical and structural characteristics [47,48]. In the SEM images of composites containing 30 phr phyto-ash, particles with varied structures were also present, dispersed relatively evenly throughout the rubber matrix. The presence of such particles indicates effective distribution within the composite, which may promote uniform reinforcement of the material. Due to their structural diversity, phyto-ash may improve mechanical properties, such as tensile strength and flexibility, as well as thermal properties, by modifying the material’s heat resistance. The varied morphology of ash makes them a promising and interesting additive for composites, potentially enhancing their functionality and durability in various applications.

In the case of plant biomass derived from beech wood, the attached SEM image revealed the presence of cellulose fiber fragments. These fibers exhibited varying lengths and thicknesses, with an elongated and thin structure, oriented in different directions. The fragmentation of these fibers during milling resulted in the formation of shorter fiber fragments. In contrast, the SEM image of the vulcanizate containing this filler showed that the fibers formed clusters within the elastomeric matrix. It was challenging to observe individual fibrous particles; instead, aggregates and agglomerates with irregular structures were evident. These observations suggest that the fibers tend to group together, likely due to physical interactions during the compounding process, and contribute to the formation of a network structure within the composite material. The irregularity of these clusters may influence the distribution and dispersion of the filler within the matrix, potentially affecting the material’s mechanical and structural properties.

In the SEM image of biochar, a characteristic porous structure is observed, with an uneven and corrugated surface. The biochar particles exhibit varying sizes and shapes, ranging from irregular fragments to more defined particles, resembling small grains. The high porosity of these particles, manifested as microscale pores and voids, indicates a large surface area, which can facilitate interactions with other materials in the composite, including the polymer matrix. This structure, characterized by microscale pores and voids, is also retained within the composites, potentially influencing their properties, such as the ability to adsorb various substances. The diverse structure of biochar, combined with its porosity, allows for effective dispersion within the elastomer, which can subsequently impact the mechanical properties of the composite, including tensile strength and elasticity.

### 3.4. Cross-Linking Density, Mechanical Properties and Hardness of the Vulcanizates

**Figure 1:**
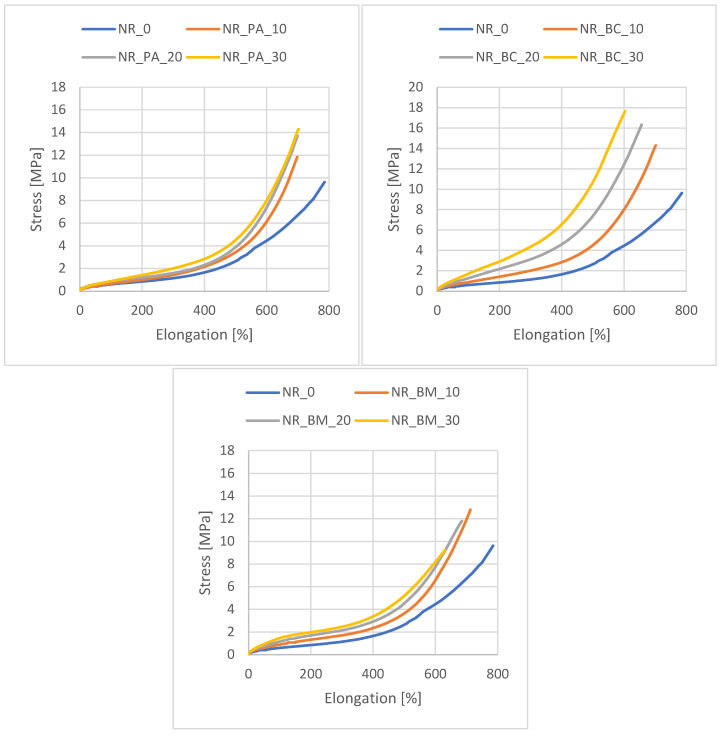
Stress–strain plots of the vulcanizates.

Table 5 shows the effect of the bioadditives used on the functional properties of the vulcanizates produced. These properties were assessed based on changes in crosslinking density (determined from equilibrium swelling tests), mechanical properties (stress at 300% of elongation), and sample hardness. The stress–strain curves are illustrated in Figure 1.

The results indicate that an increase in filler content leads to a corresponding rise in cross-linking density, corroborating previous observations of enhanced torque and vulcanization enthalpy. The highest cross-linking densities, indicative of the most extensive spatial networks, were observed for biochar and wood biomass. These findings are consistent with the ΔM parameters and enthalpy values. This confirms that plant biomass and biochar exhibit the highest activity in promoting cross-linking processes, resulting in the formation of stable bonds and dense cross-linked networks. In contrast, mixtures containing phyto-ash exhibited a cross-linking density (Ve) higher than the reference sample but slightly lower than those containing biochar and wood fibers. In phyto-ash, in addition to magnesium and zinc compounds, which can play an important role in cross-linking processes by activating vulcanization mechanisms, other metals, such as iron and copper, are also present. These elements have the ability to catalyze degradation processes, particularly the decomposition of sulfide bonds, which are responsible for the stability of the cross-linked structure of elastomers [49,50,51]. As a result, in composite systems containing phyto-ashes, competing processes can occur simultaneously—on the one hand, an increase in the degree of cross-linking, and on the other, the degradation of cross-linking bonds caused by these metals. The simultaneous occurrence of cross-linking and degradation processes can lead to a weakening of the spatial structure of elastomeric composites. Consequently, in the case of natural rubber modified with phyto-ashes, lower cross-linking density values are observed compared to systems without this additive, which may affect its mechanical properties and operational durability.

Hardness results further validate the relationship between filler content and cross-linking density. All samples containing fillers demonstrated higher hardness compared to the reference sample, with hardness increasing proportionally to the filler content in the polymer mixture. This effect was particularly pronounced in samples with biochar and wood biomass, likely due to the inherent hardness of these bioadditives. Higher hardness values were associated with increased cross-linking densities, contributing to improved mechanical properties and more robust vulcanizate structures.

The analysis of the SE300 parameter for composites containing beech wood bioadditives enables the assessment of their mechanical properties in terms of the potential to enhance the material structure. It has been shown that at low deformations, biomass and biochar exhibit a significant strengthening effect, reflected in increased SE300 parameter values. In contrast, composites filled with beech wood ash show slightly lower values of this parameter, which may result from the different structural properties of the fillers used. The structure of the composites plays a crucial role in determining their mechanical properties. An important factor influencing the results is the network bonds within the material and the presence of the secondary structure of the filler in the polymer matrix. Scanning electron microscopy (SEM) analysis revealed that biochar has a porous structure and a large specific surface area, which may promote the formation of additional physical interactions, thereby enhancing the mechanical properties of the composite. Stress–strain curve analysis indicated that composites containing ash exhibited slightly higher elongation values compared to the other tested samples. This correlates with the results of cross-linking density, which was marginally lower for these samples. The tensile strength (TS) values for these composites ranged from 12 to 14 MPa. Furthermore, it was observed that the tensile strength increased slightly with increasing filler content. In contrast, composites based on rubber containing biomass showed the lowest tensile strength values, which can be attributed to the specific structure of the filler. The ground beech wood particles, which were larger in size, likely formed larger clusters, weakening the structural integrity of the material and contributing to premature failure of the sample. This phenomenon is consistent with the lowest elongation values observed for these vulcanizates. An increase in the content of this additive led to a decrease in the tensile strength (TS) value.

For biochar, the strengthening effect at low strains was also evident in the mechanical properties of the composites. Samples containing biochar exhibited tensile strength (TS) values ranging from 15 to 18 MPa, with these values depending on the amount of biofiller used. A likely factor influencing the obtained results is the specific structure of the composites and the interactions between the components of the dispersed phase and the polymer matrix.

### 3.5. Thermogravimetric Analysis of the Biomass and Vulcanizates

The parameters obtained from thermogravimetric curves of biocomposites and beech wood are shown in Table 6. Vulcanizates with the addition of biochar and phyto-ash were characterized by increased thermal resistance. This is due to the fact that the fillers used were processed at temperatures higher than the decomposition of the rubber itself, which consequently improved the thermal stability of the composite.

In the case of the addition of wood flour composed mainly of lignin, cellulose and hemicellulose, i.e., compounds that decompose at temperatures of 180–500 °C, a significant decrease in the thermal stability of the composite with its addition could be observed.

In the study of the base beech sawdust from which all bioadditives were made, it can be observed that the material itself is not thermally stable.

The thermogravimetric analysis (TGA) method enables the recording of mass losses in composite materials across specific temperature ranges. This is determined based on the correlation between the rubber content and the thermal degradation behavior of the composite.

Based on the results, the highest mass loss was observed for vulcanizates containing biochar, as the filler underwent combustion at the analyzed temperatures. This suggests that biochar contributes significantly to the thermal degradation process within this range.

Slightly lower mass losses were recorded for composites containing phyto-ash and wood biomass. This can likely be attributed to the combustion of carbonaceous components present in these fillers, which influence the overall decomposition process. The reduced degradation rate compared to biochar-containing samples may indicate a higher proportion of thermally stable inorganic components. Reduced thermal stability of biocomposites was observed in rubber containing plant biomass, primarily due to the low thermal resistance of the lignocellulosic filler (as confirmed by TGA tests for beech wood, where T_5_ was approximately 270 °C), its potential to initiate oxidative degradation, and unfavorable effects such as moisture retention and low thermal conductivity.

The highest solid residue was found in composites with phyto-ash. This is likely due to the fact that phyto-ash undergoes high-temperature processing during its preparation, which enhances its thermal stability. As a result, a substantial fraction of the filler remains unburned after the thermal analysis. A relatively high residual mass was also observed for samples containing biochar. This could be explained by the formation of phyto-ash-like structures during combustion, which exhibit resistance to further degradation at the tested temperatures.

In contrast, the lowest solid residue was obtained for composites containing wood flour. This is expected, as wood flour is the least thermally stable filler among those analyzed. Its primary organic composition leads to extensive thermal decomposition, resulting in minimal residual mass.

### 3.6. The Influence of Thermo-Oxidative Aging on Selected Functional Properties of Biocomposites

**Figure 2:**
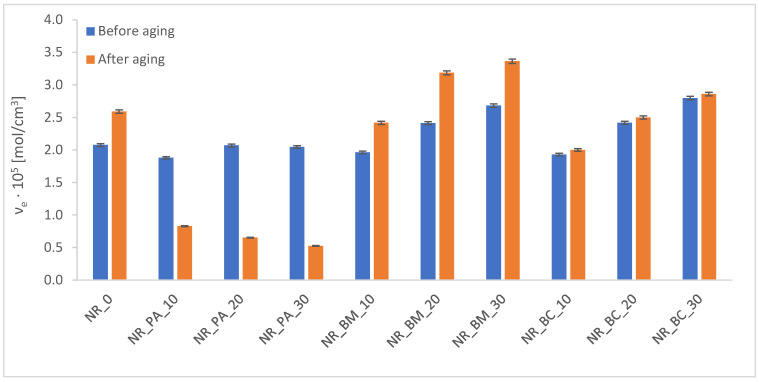
Influence of thermo-oxidative aging on crosslinking density of vulcanizates.

**Figure 3:**
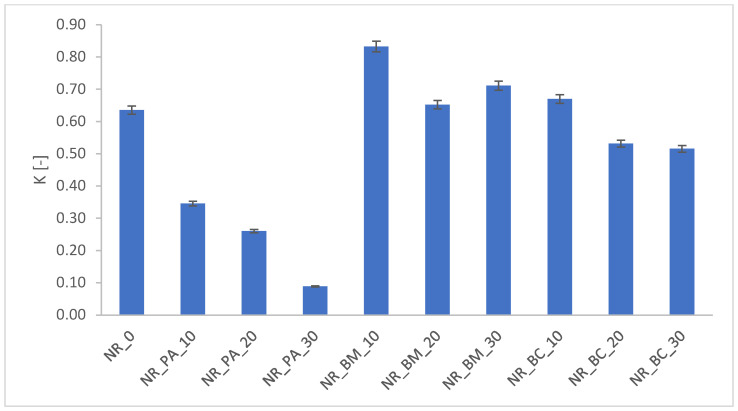
Aging factor of biocomposites subjected thermo-oxidative aging.

**Figure 4:**
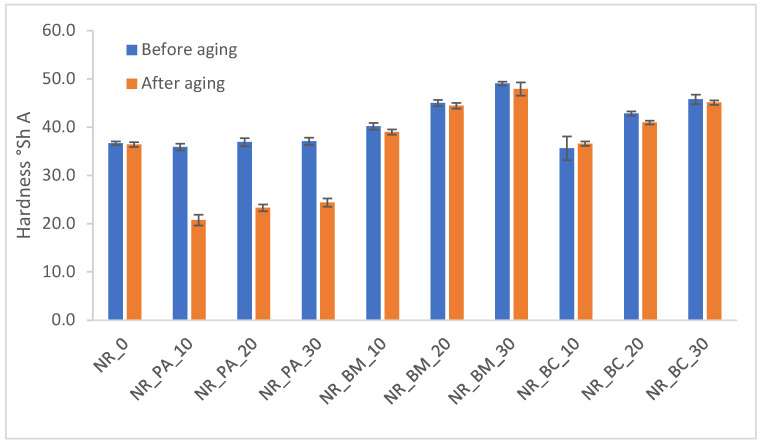
Influence of thermo-oxidative aging on hardness of vulcanizates.

During service, rubber products are subjected to a variety of environmental factors that contribute to their degradation. Elevated temperatures and ultraviolet radiation initiate the aging processes in rubber vulcanizates; therefore, it is essential to examine how various factors—such as modifications in the composition of rubber mixtures—affect the material’s resistance to these processes. The effects of aging were evaluated by analyzing changes in selected functional properties of the tested materials, including crosslink density (Figure 2), mechanical properties (Figure 3), hardness (Figure 4), and the contact angle of the vulcanizates (Table 7) following thermo-oxidative aging.

In examining the cross-link density of vulcanizates after aging, three probable reactions in elastomeric materials can be identified. The first is catalytic degradation, which involves the presence of substances that catalyze chain degradation and oxidation processes. These substances can accelerate the breakdown of polymer chains, negatively affecting the material’s performance over time. The second reaction is secondary cross-linking, which refers to reactions that may occur due to the incomplete utilization of cross-linking groups during vulcanization. This can potentially lead to an increase in cross-link density, which may affect the material’s mechanical properties and stability. Lastly, radical scavenging occurs when active substances, often derived from biomass, capture free radicals, thereby delaying or preventing harmful degradation processes. This reaction helps preserve the material’s integrity by inhibiting the further breakdown of polymer chains. These three reactions together play a crucial role in determining the long-term performance of vulcanized elastomeric materials after aging.

Based on the results obtained via the equilibrium swelling method, samples containing phyto-ash exhibited high degradation of cross-links after aging. This degradation likely arises from disordered degradation reactions catalyzed by variable-valence metals present in the phyto-ash, which facilitate the degradation of polymeric materials. In contrast, wood biomass appears to contain active antioxidant substances—such as phenolic acids or other phenolic compounds—that delay degradation. Additionally, the lignocellulosic biomass present in wood flour may also contribute to its anti-aging effect. For samples incorporating biochar, secondary cross-linking processes may occur, potentially due to incomplete cross-linking during the vulcanization process.

The resistance of vulcanizates to aging is quantified by the aging coefficient K. A value of K approaching unity indicates a higher resistance to aging processes, as it reflects smaller changes in the mechanical properties of the vulcanizates. According to the calculated K values presented in Figure 3, thermo-oxidative aging exerted a variable impact on the degradation of the vulcanizates. This finding is corroborated by the observed effects on the cross-linking density of the composites. Moreover, both mechanical testing and hardness after aging revealed that composites incorporating biochar and wood biomass experienced the least degradation, whereas those containing phyto-ash exhibited the most significant deterioration.

The last stage of the research concerning the influence of aging processes on the change in functional characteristics of vulcanizates was to determine the hydrophobicity or hydrophilicity of composites by measuring the contact angle. Pictures of the drops being deposited, together with the determined values of the contact angles, were gathered and placed in Table 7.

In surface wettability studies using contact angle measurements after simulated thermo-oxidative aging of vulcanized materials, changes in surface hydrophobicity were observed. In the case of biomass and biochar composites, aging resulted in an increase in surface hydrophobicity, as confirmed by a higher contact angle. This behavior can be explained by several factors. First, the aging process likely led to the degradation of polar functional groups, such as hydroxyl or carboxyl groups, on the surface of the vulcanizates. The reduction in these hydrophilic groups decreased their affinity for water molecules, thus increasing the surface hydrophobicity. Furthermore, as a result of aging, the polymer matrix may have undergone slight network densification, leading to a more compact cross-linked structure, reducing surface polarity and increasing hydrophobicity. An increase in cross-link density was observed in network structure measurements, indicating that the vulcanizates experienced strengthening, which additionally contributed to the increased water resistance. Moreover, due to reactions with the environment, such fillers may migrate to the surface, creating a layer with higher hydrophobicity. In the case of composites containing ash, which were significantly degraded, a decrease in cross-link density was observed, correlating with the breakdown of sulfur bonds in the polymer network. This degradation may have led to the formation of more polar functional groups on the surface, increasing its affinity for water and making the surface more hydrophilic. As a result, after aging, the contact angle decreased, indicating that the surface became more susceptible to water wetting. The decrease in cross-link density likely led to a less stable network structure, which further contributed to the increase in surface hydrophilicity. These results suggest that the degree of degradation and changes in network structure during aging play a key role in determining surface properties, with degradation leading to more hydrophilic surfaces and the formation of a more densely cross-linked structure resulting in hydrophobic behavior.

## 4. Conclusions

The study shows that adding phyto-ash slightly reduced minimum torque, correlating with lower viscosity due to its high mineral content. In contrast, biochar and biomass increased G_min_, suggesting enhanced stiffness. Among the bio-composites, biomass-filled mixtures exhibited the highest maximum torque (4.41–5.92 dNm), likely due to filler cohesion, while phyto-ash composites had the lowest G_max_ (4.00–4.60 dNm), indicating reduced cross-linking density.

Vulcanization times varied as phyto-ash and biomass composites had shorter or comparable curing times, while biochar extended t_90_, possibly due to filler absorption of curing agents. Phyto-ash composites showed the shortest curing times (2.02–2.28 min), likely due to basic compounds accelerating vulcanization, whereas biochar, with its acidic nature, delayed the process (2.39–2.44 min).

Differential scanning calorimetry indicated a slight decrease in glass transition temperature (Tg) with bio-fillers, likely due to increased polymer chain mobility. Biochar, being non-polar, may reduce van der Waals forces, further lowering Tg. Increased vulcanization enthalpy suggests stronger polymer-filler interactions and higher cross-linking density, influenced by catalytic metal components.

Scanning electron microscopy revealed diverse morphologies in phyto-ash, a porous structure in biochar, and cellulose fiber aggregates in biomass. Cross-linking density was higher in biochar and biomass (2.68 × 10^−5^ mol/cm^3^ and 2.77 × 10^−5^mol/cm^3^, respectively) composites, whereas phyto-ash samples had lower (2.05 × 10^−5^ mol/cm^3^) cross-linking densities, likely due to metal-catalyzed degradation.

Hardness tests indicated that all bio-filled samples were stiffer than the reference, with biochar and biomass composites being the hardest. Thermogravimetric analysis revealed improved thermal stability for biochar and phyto-ash, while biomass composites exhibited lower resistance due to lignin and cellulose degradation.

Thermo-oxidative aging analysis showed that phyto-ash composites degraded the most, likely due to transition metal-catalyzed sulfur bond breakdown. Biochar and biomass composites exhibited better aging resistance. Contact angle measurements indicated increased hydrophobicity in aged biochar and biomass composites due to degradation of polar groups and higher cross-linking density.

The findings of this study highlight the potential of bio-fillers as sustainable and functional reinforcements in elastomer composites. Due to their ability to modify mechanical properties, biochar and biomass-filled composites could be utilized in applications requiring enhanced stiffness and durability, such as automotive components, industrial seals, or vibration-damping materials. The improved thermal stability of biochar and phyto-ash composites suggests their suitability for high-temperature environments. However, further research is needed to optimize filler dispersion and interfacial bonding to enhance overall performance. Investigating hybrid filler systems combining biochar, biomass, and phyto-ash could provide insights into synergistic effects on mechanical and aging properties. Additionally, studying the influence of surface modifications on bio-fillers could improve their compatibility with elastomers, leading to better cross-linking efficiency and mechanical performance. Future work should also focus on the long-term environmental impact and recyclability of these composites, ensuring their viability as eco-friendly alternatives in sustainable material development.

## Figures and Tables

**Table 1:** Composition of rubber mixtures.

Sample	NR [phr]	Filler [phr]		
		Phyto-Ash	Biochar	Biomass
NR_0	100	-	-	-
NR_PA_10		10		
NR_PA_20		20		
NR_PA_30		30		
NR_BC_10			10	
NR_BC_20			20	
NR_BC_30			30	
NR_BM_10				10
NR_BM_20				20
NR_BM_30				30

PA—phyto-ash, BC—biochar, BM—biomass.

**Table 2:** Rheometric properties of elastomer mixtures (standard deviations: G_min_ ± 0.1 dNm, G_max_ ± 0.7 dNm ∆G ± 0.5 dNm, t_5_ ± 0.2 min., t_90_ ± 0.4 min).

Sample	G_min_ [dNm]	G_max_ [dNm]	ΔG [dNm]	t_5_ [min]	t_90_ [min]
NR_0	0.40	3.96	3.56	1.11	1.95
NR_PA_10	0.32	4.00	3.68	0.93	2.04
NR_PA_20	0.32	4.17	3.85	0.85	2.13
NR_PA_30	0.31	4.60	4.29	0.77	2.28
NR_BC_10	0.43	3.80	3.37	1.34	2.44
NR_BC_20	0.48	4.37	3.89	1.29	2.58
NR_BC_30	0.42	5.48	5.06	0.95	2.39
NR_BM_10	0.45	4.41	3.96	1.04	2.08
NR_BM_20	0.45	4.89	4.44	1.23	2.79
NR_BM_30	0.47	5.92	5.45	0.89	2.12

**Table 3:** Thermal characteristics of elastomeric compounds determined by DSC analysis.

Sample	Vulcanization Enthalpy [J/g]	Initial Vulcanization Temperature [°C]	Final Vulcanization Temperature [°C]	Glass Transition Temperature (Tg) [°C]
NR_0	8.6	150	227	−62.8
NR_BM_30	10.0	137	226	−63.3
NR_BC_30	15.8	132	231	−63.5
NR_PA_30	14.2	137	238	−64.2

**Table 4:** SEM images of samples.

Filler	NR Composites
PA	NR_PA_30
** 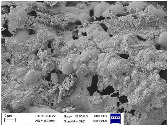 **	** 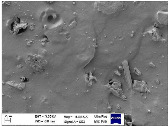 **
BM	NR_BM_30
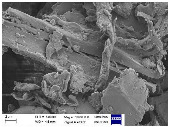	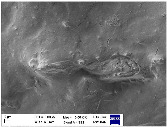
BC	NR_BC_30
** 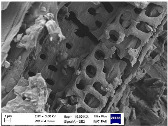 **	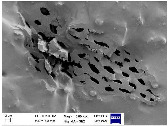

**Table 5:** Crosslinking density, tensile strength, stress at 300% of elongation and hardness of the biocomposites.

Sample	V_e_*10^5^	SE_300_	Hardness
	[mol/dm^3^]	[Mpa]	[°Sh A]
NR_0	2.08 ± 0.02	1.28 ± 0.18	36.6 ± 0.4
NR_PA_10	1.88 ± 0.01	1.41 ± 0.11	35.8 ± 0.7
NR_PA_20	2.07 ± 0.03	1.60 ± 0.01	36.9 ± 0.7
NR_PA_30	2.05 ± 0.02	1.51 ± 0.05	37.1 ± 0.9
NR_BM_10	1.96 ± 0.01	1.70 ± 0.02	40.2 ± 0.7
NR_BM_20	2.41 ± 0.01	2.12 ± 0.05	45.0 ± 0.7
NR_BM_30	2.68 ± 0.02	2.47 ± 0.03	49.1 ± 0.4
NR_BC_10	1.93 ± 0.03	2.02 ± 0.04	39.6 ± 1.2
NR_BC_20	2.42 ± 0.03	3.13 ± 0.03	42.9 ± 0.4
NR_BC_30	2.77 ± 0.02	4.36 ± 0.06	45.8 ± 0.9

**Table 6:** Thermal stability of biomaterials.

Sample	T_5_ [°C]	T_50_ [°C]	Mass Loss 1 (25–600 °C) [%]	Mass Loss 2 (600–900 °C) [%]	Solid Residue [%]
NR_0	349	404	88.0	10.8	1.2
NR_BM_30	296	398	87.2	4.5	7.1
NR_BC_30	352	415	73.5	17.2	9.0
NR_PA_30	348	408	74.1	3.6	22.1
Beech wood	269	369	78.7	17.3	4.0

T_5_—temperature of 5% mass loss [°C]; T_50_—temperature of 50% mass loss [°C].

**Table 7:** Influence of thermo-oxidative aging on hydrophobic properties of vulcanizates. The standard deviation of the contact angle measurements did not exceed 5%.

Before Aging	After Aging
NR_0	
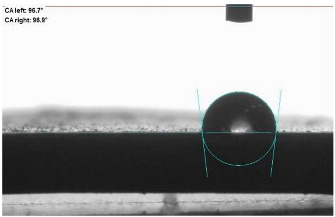	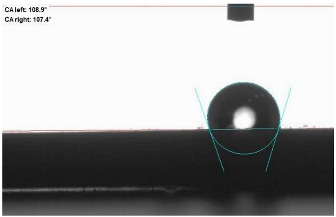
NR_BM_10	
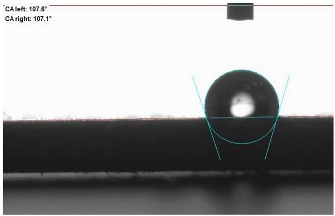	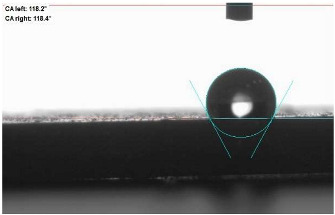
NR_BM_20	
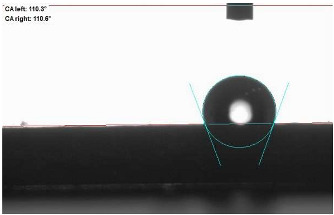	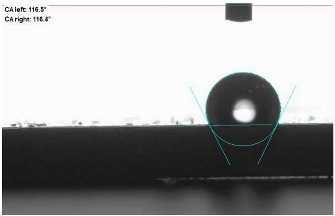
NR_BM_30	
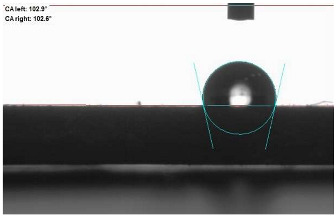	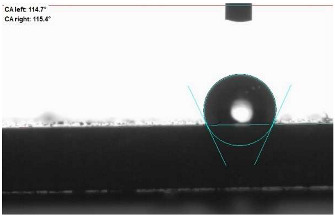
NR_PA_10	
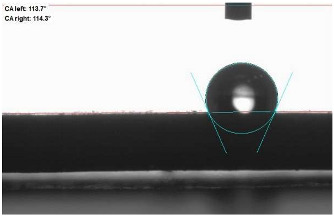	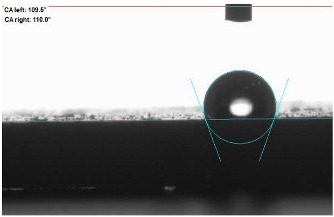
NR_PA_20	
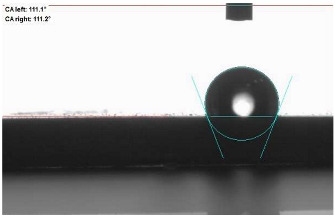	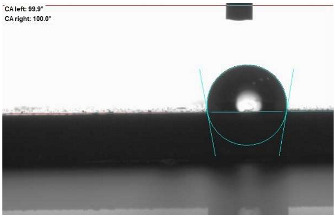
NR_PA_30	
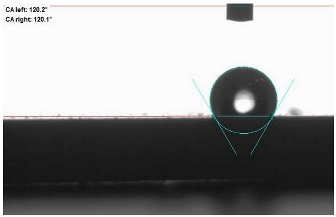	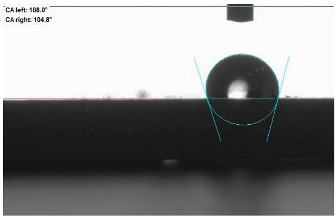
NR_BC_10	
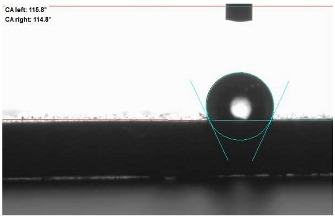	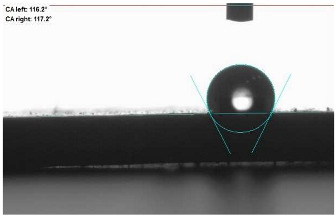
NR_BC_20	
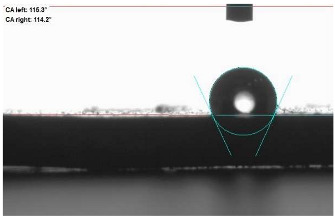	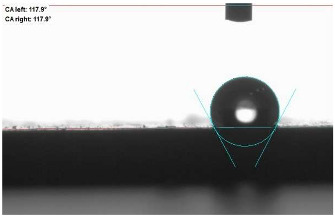
NR_BC_30	
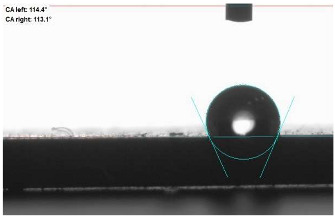	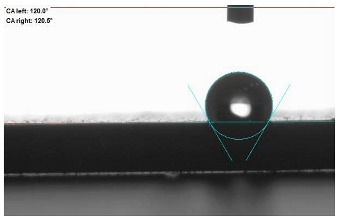

## Data Availability

The original contributions presented in the study are included in the article, further inquiries can be directed to the corresponding authors.
